# Plasma and interstitial levels of endocannabinoids and N-acylethanolamines in patients with chronic widespread pain and fibromyalgia: a systematic review and meta-analysis

**DOI:** 10.1097/PR9.0000000000001045

**Published:** 2022-11-07

**Authors:** Inna Kurlyandchik, Romy Lauche, Evelin Tiralongo, Leon N. Warne, Janet Schloss

**Affiliations:** a National Centre for Naturopathic Medicine, Southern Cross University, Lismore, New South Wales, Australia; b School of Pharmacy and Medical Sciences, Griffith University, Gold Coast, Queensland, Australia; c Little Green Pharma, West Perth, Western Australia, Australia; d College of Science, Health, Engineering and Education, Murdoch University, Perth, Western Australia, Australia; e School of Pharmacy and Biomedical Sciences, Curtin Health Innovation Research Institute, Curtin University, Perth, Western Australia, Australia

**Keywords:** Fibromyalgia, Chronic widespread pain, Endocannabinoid system, Endocannabinoids, N-acylethanolamines

## Abstract

The endocannabinoid system (ECS) is an essential endogenous signaling system that may be involved in the pathophysiology of chronic widespread pain (CWP) and fibromyalgia syndrome (FMS). Further research is required to understand the role of ECS in the development and maintenance of CWP and FMS. We provided the first systematic review and meta-analysis exploring the clinical relevance of ECS alterations in patients with CWP and FMS by comparing plasma and interstitial levels of endocannabinoids and N-acylethanolamines in patients and healthy controls. A systematic search was conducted to identify studies that measured plasma and/or interstitial levels of endocannabinoids and N-acylethanolamines in patients with CWP or FMS and healthy controls. A total of 8 studies were included for qualitative review, and 7 studies were included for meta-analysis. The findings identified increased plasma levels of oleoylethanolamide and stearoylethanolamide in patients with FMS compared with those in controls (*P* = 0.005 and *P* < 0.0001, respectively) and increased plasma levels of palmitoylethanolamide and interstitial levels of stearoylethanolamide in patients with CWP compared with those in controls (*P* = 0.05 and *P* = 0.001, respectively). There were no significant differences in other ECS parameters. Most studies did not account for variables that may influence ECS function, including cannabis use, concomitant medication, comorbidities, physical activity, stress levels, circadian rhythm, sleep quality, and dietary factors, suggesting that future studies should explore the correlation between these variables and endocannabinoid activity. We highlight the importance of investigating endocannabinoid activity in CWP and FMS because it will underpin future translational research in the area.

## Abstract 2

Supplemental Digital Content is Available in the Text.

Increased levels of plasma oleoylethanolamide and stearoylethanolamide in patients with fibromyalgia and plasma palmitoylethanolamide and interstitial stearoylethanolamide in patients with chronic widespread pain suggest that the endocannabinoid system may be dysregulated in these conditions.

## 1. Introduction

Fibromyalgia syndrome (FMS) is manifested by chronic widespread pain (CWP) as the cardinal symptom and other somatic and psychological impairments, including morning stiffness, severe fatigue, sleep disturbances, cognitive dysfunction, depression, and anxiety.^7,51^ Therefore, the terms fibromyalgia and CWP are often incorrectly used interchangeably. Chronic widespread pain may also occur as a symptom of other conditions, including musculoskeletal, metabolic, endocrine, psychological, neurological, and medication-related disorders.^20^ Failure to recognize other conditions that can masquerade as FMS could adversely affect patient outcomes.^20^ Chronic widespread pain and FMS are relatively common, affecting 2% to 5% of developed countries' populations.^51^ The most common criteria for diagnosis of FMS are the 1990 American College of Rheumatology (ACR) criteria, where CWP is defined as “pain lasting >3 months that affects both sides of the body both above and below the waist, including some part of the axial skeleton.”^66^ The ACR criteria were revised in 2010 and 2016 with the latest CWP definition standardized to pain in at least 4 of 5 pain regions.^65^ The heterogeneity of CWP and FMS risk factors and pathophysiological mechanisms requires individualized treatment strategies, with most having only moderately effective outcomes.^40^

The endocannabinoid system (ECS) is an essential endogenous signaling system involved in various physiological processes, including regulation of pain, inflammation, sleep, cognition, and energy metabolism.^2,3,35,53,67^ According to the classical definition, the ECS is composed of the type-1 and type-2 cannabinoid receptors (CB_1_ and CB_2_), endogenous cannabinoid receptor ligands (endocannabinoids), and enzymes responsible for endocannabinoid biosynthesis.^12^ A broader definition of the ECS also includes endocannabinoid-like lipid mediators, which belong to the same chemical class as the endocannabinoids (ie, amides, esters, and ethers of long-chain fatty acids) but bind to different receptors.^12,61^

Type-1 cannabinoid receptors are expressed mainly in the brain and, to a lesser extent, in the peripheral tissues, whereas CB_2_ receptors are primarily present in immune cells and tissues.^38^ The main endocannabinoids N-arachidonoylethanolamide (AEA or anandamide) and 2-arachidonoylglycerol (2-AG) bind to CB_1_ and CB_2_ receptors.^3^ Anandamide behaves as a partial agonist of CB_1_ but is virtually inactive at CB_2_, whereas 2-AG is a full agonist of CB_1_ and CB_2_.^33^ Therefore, AEA and 2-AG elicit various biological effects as cannabinoid receptor ligands, including the cannabinoid tetrad characterized by hypolocomotion, hypothermia, catalepsy, and analgesia.^61^ They also induce decreases in heart rate, blood, and intraocular pressure^61^ and show anti-inflammatory and immunomodulatory activity.^62^ Anandamide has also been shown to activate vanilloid receptors; however, its physiologic role as a vanilloid receptor agonist is not fully understood.^11^

Endocannabinoid-like lipid mediators such as N-acylethanolamines (NAEs), including palmitoylethanolamide (PEA), oleoylethanolamide (OEA), and stearoylethanolamide (SEA), exert their biological effects potentially through molecular targets other than cannabinoid receptors.^61^ Palmitoylethanolamide selectively activates peroxisome proliferator-activated receptor-alpha (PPAR-α) and G protein–coupled receptor 55 (GPR55)^29,37^ and demonstrates analgesic, anti-inflammatory, antiallergic, and neuroprotective properties.^1^ Oleoylethanolamide suppresses appetite, regulates food intake and body weight, and shows analgesic and anti-inflammatory properties using different mechanisms through activation of PPAR-α, GPR119, and vanilloid receptor 1 (TRPV1).^29^ Stearoylethanolamide demonstrates anti-inflammatory and neuroprotective properties,^32^ potentially through activation of PPAR-γ,^5^ although its molecular targets have yet to be fully elucidated.

In human studies, endocannabinoids and NAEs have been extracted from many biological fluids and tissues, including plasma and interstitial microdialysates, and quantified using various liquid chromatography methods coupled with mass spectrometry.^68^ Recently, research efforts have been devoted to understanding the relationship between ECS and chronic pain conditions, including FMS, suggesting that endocannabinoid deficiency could underpin the pathophysiology of such disorders.^47–49^ Therefore, further research is required to better understand the mechanisms of the ECS and implications for effectively treating CWP and FMS.

To date, it has not been established whether the endocannabinoid activity in patients with CWP and FMS correlates with the clinical status of the conditions. We explored this question with a systematic review and meta-analysis of the available evidence and investigated the clinical relevance of ECS alterations in patients with CWP and FMS by comparing the differences in plasma and interstitial levels of endocannabinoids and NAEs between patients and healthy controls.

## 2. Methods

The systematic review and meta-analysis were conducted in accordance with the Preferred Reporting Items for Systematic Reviews and Meta-Analyses (PRISMA) guidelines (Page et al., 2021). The protocol was registered in PROSPERO (CRD42021272567). The full search strategy can be found in the Supplementary Information—Research Protocol (available at http://links.lww.com/PR9/A177).

The systematic search was conducted by author I.K. from August 16, 2021, to August 18, 2021, using electronic databases MEDLINE (PubMed), CINAHL (Ebsco), PsycINFO (Ebsco), and Scopus (Elsevier) to identify publications reporting plasma and/or interstitial levels of endocannabinoids and NAEs in patients with CWP and FMS.

### 2.1. Inclusion criteria

The inclusion criteria were studies reporting plasma and/or interstitial levels of endocannabionoids (anandamide, 2-arachidonoylglycerol) and N-acylethanolamines (palmitoylethanolamide, oleoylethanolamide, stearoylethanolamide) in patients with clinically diagnosed CWP or FMS when compared against healthy controls.

### 2.2. Exclusion criteria

(1) In vitro and animal studies.
(2) Systematic reviews or meta-analyses.
(3) Studies lacking numerical data.
(4) Language other than English, German, or Russian.

### 2.3. Search terms

(1) “Chronic widespread pain” OR fibromyalgia.
(2) Endocannabinoid OR Anandamide OR N-arachidonoylethanolamide OR N-acylethanolamine OR Arachidonoylethanolamide OR 2-arachidonoylglycerol OR oleoylethanolamide OR palmitoylethanolamide OR stearoylethanolamide.
(3) 1 AND 2.

Search results were limited to human studies. Titles and abstracts of retrieved publications were imported into an EndNote Library. Duplicates were identified and removed. Studies were then analyzed by screening through titles and then abstracts by authors I.K. and J.S. Studies that clearly did not satisfy the inclusion criteria were excluded; full texts of the remaining studies were obtained. After reading the full texts, an agreement was reached between all review authors on a final selection of reports to be included. Author I.K. extracted data from all included studies into an electronic summary table, which was reviewed by author J.S.

Two reviewers (authors J.S. and I.K.) independently assessed the quality of the included studies using the modified Newcastle-Ottawa scale and through this process, achieved identical scores in every category, as well as overall (full consensus) (Table 1).

**Table 1:** Quality assessment of included studies based on the Newcastle-Ottawa scale.

	Selection				Comparability		Outcome		Overall score (out of 8)
	Adequate case definition	Representativeness of the sample	Selection of controls	Definition of controls	Age	Sex	Assessment of the outcome	Statistical test	
Baumeister et al.^4^	1	1	1	1	0	0	1	1	6
Ghafouri et al.^16^	1	1	1	1	1	1	1	1	8
Hellström et al.^21^	1	1	1	1	0	1	1	1	7
Kaufmann et al.^34^	1	1	1	1	1	1	1	1	8
Stensson et al.^56^	1	1	1	1	0	1	1	1	7
Stensson et al.^55^	1	1	1	1	1	1	1	1	8
Stensson et al.^57^	1	1	1	1	1	1	1	1	8
Stensson et al.^54^	1	1	1	1	1	1	1	1	8

Adapted version of the Newcastle-Ottawa quality assessment scale for case-control studies:

Selection (max 4 points):

(1) Is the case definition adequate? (1) yes, with independent validation (American College of Rheumatology 1990) (1 point); (2) yes, eg, record linkage or based on self-reports;and (3) No description.

(2) Representativeness of the sample: (1) consecutive or obviously representative series of cases (1 point); (2) potential for selection biases or not stated.

(3) Selection of controls: (1) community controls (1 point); (2) hospital controls; and (3) no description.

(4) Definition of controls: (1) no history of disease (1 point); (2) no description of source.

Comparability (max 2 points):

(1) Comparability of cases and controls based on the design or analysis: (1) study controls for age (1 point); (2) study controls for sex (1 point).

Outcome (max 2 points):

(1) Assessment of the outcome: (1) objective measure (1 point); (2) validated self-report measures (1 point); and (3) no information or nonvalidated measures.

(2) Statistical test: (1) The statistical test used to analyze the data is clearly described and appropriate, and the measurement of the association is presented, including confidence intervals and/or the probability level (*P* value) (1 point); (2) the statistical test is not appropriate, not described, or incomplete.

### 2.4. Outcomes and meta-analyses

We investigated the clinical relevance of ECS alterations in patients with CWP and FMS by comparing the differences in plasma and interstitial levels of endocannabinoids and NAEs between patients and healthy controls.

A quantitative analysis of the differences in ECS markers between patients and healthy controls was performed when 2 or more studies were available in each subgroup (FMS/CWP). Median values, interquartile ranges, standard errors, and confidence intervals (CIs) were converted to mean values and SDs following validated protocols.^22,28^

All meta-analyses were conducted in RevMan, version 5.4.^59^ The standardized mean difference (SMD) was used as summary statistics in meta-analyses. Because meta-analyses of observational studies are typically characterized by significant heterogeneity, we pooled data using random-effects models.^42^ Statistical significance between patients and controls was assessed with a *z* test, where *P* < 0.05 was considered statistically significant.

We assessed heterogeneity with the Cochrane Q test, where *P* < 0.10 represents statistically significant between-study heterogeneity, and the degree of heterogeneity was quantified with the *I*^*2*^ index, where *I*^*2*^ values above 25%, 50%, and 75% represent low, moderate, and high heterogeneity, respectively.^23,24^ Sensitivity analyses were performed using the “leave-one-out” method by removing a single study each time and repeating the analysis to estimate the effect of any individual study on the overall SMD.

To identify factors affecting the levels of endocannabinoids and NAEs, qualitative data were collected, including the sampling, analytical methods and diagnostic criteria used, the number of participants, their age, sex, body mass index (BMI), medication use, cannabis use, comorbidities, the intensity of pain, and depression and anxiety levels.

## 3. Results

**Figure 1.**
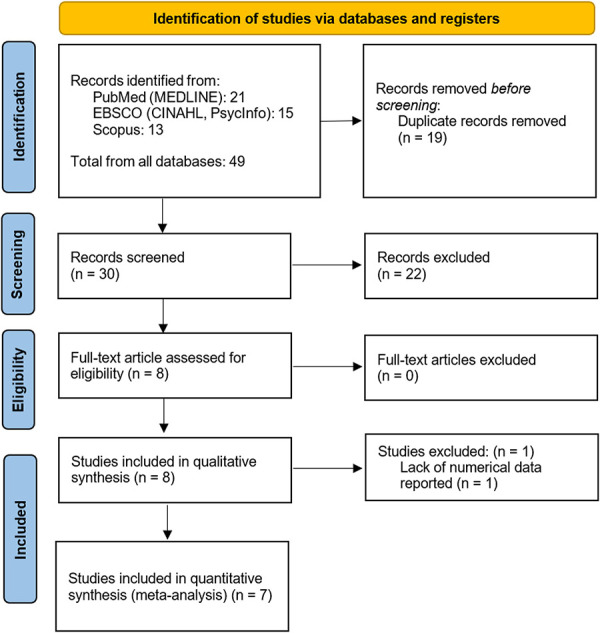
Preferred reporting items for systematic reviews and meta-analyses flow diagram of study selection process.

The initial database search identified 49 citations. After removing duplicates and screening titles, abstracts, and full texts, a total of 8 studies met the inclusion criteria for qualitative analysis.^4,16,21,34,54–57^ Of those 8 studies, 1 was excluded from meta-analysis because of lack of numerical data reported, leaving a total of 7 studies eligible for meta-analysis.^4,16,21,54–57^ See the PRISMA diagram in Figure 1.

The characteristics of included studies are summarized in Table 2. Four articles investigated the ECS markers in patients with FMS^4,34,54,57^ and 4 studied CWP cohorts.^16,21,55,56^ One of the CWP studies reported morning and afternoon data^21^ with the data captured from separate sample groups (morning and afternoon); therefore, we included these study results as 2 individual reports. Two studies collected samples before and after exercise: one investigated plasma levels in patients with FMS^54^ and the other studied interstitial levels in patients with CWP.^16^ No other study reported data on the changes of ECS markers in response to exercise; therefore, only data collected before exercise were included in the meta-analyses.

**Table 2:** Main characteristics of studies included in qualitative analysis.

First author (year)	No. of participants	Diagnostic criteria	ECS markers studied	Sampling/analytical method/s	Age, y	Male/female	BMI, kg/m^2^	Cannabis use	Medication use	Comorbidities	Intensity of pain, depression and anxiety	Other blood markers	Main outcomes			
Baumeister (2018)	n = 89 (FMS) n = 36 (HC)	ACR 1990	Plasma AEA, 2-AG, PEA	Venepuncture/liquid chromatography/multiple reaction monitoring (LC/MRM)	FMS: 55.4 (9.5) HC: 61.4 (12.4)	FMS: 7/82 HC: 20/16	FMS: 29.2 (6.5) HC: 26.9 (4.0)	Cannabis users were excluded from the study	Antidepressants: FMS: 41.6% (38) HC: 0% (0), *P* = 0.001 Opiates: FMS: 5.6% (6) HC: 0% (0), *P* = 0.1 NSAIDs: FMS: 58.4% (53) HC: 13.9% (5), *P* = 0.001 Channel blockers: FMS: 15.7% (14) HC: 0% (0), *P* = 0.01	Diabetes mellitus: FMS: 11.2% (10) HC: 2.8% (1), *P* = 0.1	Average pain/4 wk (NRS 0–10): FMS: 6.2 (1.6) HC: 0.0 (0.0), *P* = 0.001 Depression (HADS): FMS: 8.1 (4.4) HC: 3.0 (3.2), *P* = 0.001 Anxiety (HADS): FMS: 9.2 (4.2) HC: 3.6 (2.5), *P* = 0.001	Not reported	FMS (pmol/L): AEA: 0.56 (0.31) 2AG: 2.18 (1.20) PEA: 5.92 (2.14)	HC (pmol/L): AEA: 0.55 (0.28) 2AG: 2.13 (1.20) PEA: 5.80 (2.53)	BF10: AEA: 0.22 2AG: 0.21 PEA: 0.24	
Kaufmann (2008)	n = 22 (FMS) n = 22 (HC)	ACR 1990	Plasma AEA	Venepuncture/high performance liquid chromatography-tandem mass spectrometry (HPLC/MS–MS)	FMS: 53.1 (2.1) HC: 48.1 (5.1)	FMS: 5/17 HC: 5/17	Not reported	Not reported	Not reported Participants had abstained from their medication for at least 24 h before blood sampling. Subjects taking immunosuppressive or acetylsalicylic acid medication were excluded	Not reported Subjects with inflammatory conditions, diabetes mellitus, endocrinologic disorders including thyroid disease, infections, hypertension, muscle or joint diseases, acute injury, pregnancy, major depressive disorder, addiction, general anxiety disorder, psychosis, major personality disorder, or current compensation claims were excluded	Pain (0–10 cm VAS): FMS: 6.4 (1.4) HC: 0.0 (0.0), *P* < 0.0001 Stress (PTSS-10): FMS: 46.7 (3.5) HC: 19.0 (2.3), *P* < 0.001	Plasma epinephrine and norepinephrine, serum cortisol, white blood cell count, neutrophil function (H_2_O_2_ production, adhesion and phagocytosis capabilities)	AEA (μg/L): FMS vs HC: *P* < 0.0001			
Stensson (2018)	n = 104 (FMS) n = 116 (HC)	ACR 1990	Plasma AEA, OEA, PEA, SEA, 2-AG	Venepuncture/liquid chromatography-tandem mass spectrometry (LC-MS/MS)	FMS: 50.8 (9.7) HC: 48.1 (5.1)	FMS: 0/104 HC: 0/116	FMS: 27.7 (5.1) HC: 24.0 (3.7)	Not reported	Not reported Participants had to refrain from analgesics, NSAIDS, or hypnotics for 48 h before examinations	Not reported Patients with FMS with high blood pressure, osteoarthritis (hip/knee), other severe somatic or psychiatric disorders, and primary causes of pain other than FMS were excluded from the study	Pain (100-mm VAS): FMS: 51.2 (21.6) HC: 2.7 (6.6) Depression (HAD-D): FMS: 7.3 (3.6) HC: 1.8 (2.4) Anxiety (HAD-A): FMS: 8.7 (4.1) HC: 3.4 (3.2)	Not reported	FMS (nM): AEA: 0.31 (0.15) OEA: 6.2 (2.3) PEA: 9.5 (2.4) SEA: 2.6 (1.0) 2AG: 14.5 (9.2)	HC (nM): AEA: 0.31 (0.15) OEA: 5.4 (1.8) PEA: 8.6 (2.5) SEA: 2.1 (0.9) 2AG: 11.1 (5.1)	*P*: AEA: 0.99 OEA: 0.006 PEA: 0.010 SEA: 0.001 2AG: 0.001	
Stensson (2020)	Primary cohort: n = 37 (FMS) n = 33 (HC)	ACR 1990	Plasma AEA, OEA, PEA, SEA, 2-AG	Venepuncture/liquid chromatography-tandem mass spectrometry (LC-MS/MS)	FMS: 50.1 (10.5) HC: 50.3 (12.8)	FMS: 0/37 HC: 0/33	FMS: 26.5 (4.7) HC: 24.4 (4.4)	Not reported	Not reported Participants had to refrain from analgesics, NSAIDS, or hypnotics for 48 h before examinations	Not reported Patients with FMS with high blood pressure, osteoarthritis (hip/knee), other severe somatic or psychiatric disorders, and primary causes of pain other than FMS were excluded from the study	Pain (100-mm VAS): FMS: 48.7 (22.9) HC: 1.9 (5.9), *P* = 0.001 Depression (HAD-D): FMS: 7.7 (4.2) HC: 3.1 (3.0), *P* = 0.001 Anxiety (HAD-A): FMS: 8.8 (4.7) HC: 1.5 (1.8), *P* = 0.001	Not reported	FMS (nM): *Pre-exercise:* AEA: 0.26 (0.13) OEA: 5.66 (1.92) PEA: 8.51 (2.03) SEA: 2.56 (1.18) 2AG: 12.8 (8.49) *Post-exercise:* AEA: 0.34 (0.18) OEA: 5.33 (2.11) PEA: 7.96 (2.69) SEA: 2.05 (1.10) 2AG: 11.5 (8.03)	HC (nM): *Pre-exercise:* AEA: 0.31 (0.16) OEA: 5.39 (1.29) PEA: 8.72 (1.63) SEA: 2.05 (0.83) 2AG: 12.5 (3.97) *Post-exercise:* AEA: 0.25 (0.14) OEA: 6.06 (1.96) PEA: 9.18 (2.54) SEA: 2.57 (1.06) 2AG: 12.6 (6.17)	*P*: *Pre-exercise:* AEA: 0.12 OEA: 0.50 PEA: 0.63 SEA: 0.04 2AG: 0.83 *Post-exercise:* AEA: 0.03 OEA: 0.14 PEA: 0.06 SEA: 0.05 2AG: 0.51	
Hellström (2016)	Morning sample: n = 5 (CWP) n = 15 (HC) Afternoon sample: n = 9 (CWP) n = 12 (HC)	ACR 1990	Plasma 2-AG, AEA, PEA, SEA, OEA, LEA	Venepuncture/Ultra-performance liquid chromatography-tandem mass spectrometry (UPLC-MS/MS)	CWP: 55.5 (6.0) HC: 47.5 (9.0)	CWP: 0/14 HC: 0/27	FMS: 26.8 (4.3) HC: 24.0 (3.0)	Not reported	Not reported All participants were asked not to use any pain medications except for paracetamol preparations 3 days before the blood sampling	Not reported Subjects with rheumatoid arthritis, systemic lupus erythematosus, Bechterew disease, multiple sclerosis, epilepsy or Parkinson disease, type I diabetes, cardiovascular disease, or endocrine diseases were excluded	Average pain/1 wk (NRS 0–10): CWP: 5.3 (1.3) HC: no pain Current pain (NRS 0–10): CWP: 3.8 (1.8) HC: no pain Depression/anxiety: Not reported	9-HODE, 13-HODE, 9,10-DiHOME, 12,13-DiHOME, 9,10,13-TriHOME, 9,12,13-TriHOME, 13-oxo-ODE, 5-HETE, 8,9-DiHETrE	CWP (nM)		HC (nM)	
													*AM:* 2AG: 3.28 (2.12) AEA: 0.16 (0.16) PEA: 2.08 (1.12) SEA: 11.31 (8.34) OEA: 1.76 (1.01) LEA: 0.62 (0.60)	*PM:* 8.30 (10.83) 0.22 (0.17) 2.96 (1.68) 10.69 (5.58) 1.29 (1.39) 0.71 (0.90)	*AM:* 5.38 (3.22) 0.18 (0.05) 1.93 (0.53) 8.44 (3.74) 0.93 (1.25) 0.62 (0.14)	*PM:* 6.39 (8.50) 0.19 (0.10) 2.73 (0.89) 11.22 (4.02) 1.77 (0.73) 0.95 (0.34)
Stensson (2017)	n = 17 (CWP) n = 21 (HC)	ACR 1990	Plasma OEA, PEA, SEA	Venepuncture/Liquid chromatography-tandem mass spectrometry (LC-MS/MS)	CWP: 41.7 (10) HC: 47.9 (9.6)	CWP: 0/17 HC: 0/21	FMS: 24.2 (2.1) HC: 26.8 (5.3)	Not reported	Not reported.	Not reported. Subjects with bursitis, disorders of the spine, tendonitis, capsulitis, postoperative conditions (neck/shoulder), prior neck trauma, neurological disease, rheumatoid arthritis or any other systemic disease, metabolic disease, malignancy, severe psychiatric illness, pregnancy were excluded.	Pain (NRS 0–10): CWP: 5.3 (2.1) HC: 0.0 (0.0), *P* < 0.001 Depression/Anxiety: Not reported	IL-6, IL-8, IL-10, TNF-α, IL-1β	CWP (nM): OEA: 11.1 (3.0) PEA: 18.1 (9.7) SEA: 38.6 (28.7)	HC (nM): OEA: 7.5 (3.7) PEA: 10.5 (6.2) SEA: 27.2 (20.7)	*P*: OEA: 0.003 PEA: 0.006 SEA: 0.164	
Ghafouri (2013)	n = 18 (CWP) n = 24 (HC)	ACR 1990	Interstitial PEA and SEA (trapezius muscle)	Microdialysis/liquid chromatography-tandem mass spectrometry (LC–MS/MS)	CWP: 47.6 (8.3) HC: 42.8 (7.3)	CWP: 0/18 HC: 0/24	FMS: 27.5 (4.0) HC: 24.3 (2.8)	Not reported	Not reported Participants were asked not to take nonsteroidal anti-inflammatory drugs for 7 d and/or paracetamol medication for 12 h before the experiment	Not reported Subjects with bursitis, disorders of the spine, tendonitis, capsulitis, postoperative conditions (neck/shoulder), prior neck trauma, neurological disease, rheumatoid arthritis or any other systemic disease, metabolic disease, malignancy, severe psychiatric illness, and pregnancy were excluded	Pain (NRS 0–10): CWP vs HC: *P* = 0.001 Depression/anxiety: Not reported	Not reported	CWP (nM): *Pre-exercise:* PEA: 1.23 (0.55) SEA: 1.48 (0.93) *Post-exercise:* PEA 0.95 (0.58) SEA: 0.95 (0.70)	HC (nM): *Pre-exercise:* PEA: 1.17 (0.84) SEA: 0.98 (0.68) *Post-exercise:* PEA: 0.85 (0.55) SEA: 0.75 (0.43)	*P*: *Pre-exercise:* PEA: NS SEA: NS *Post-exercise:* PEA: NS SEA: NS	
Stensson (2016)	n = 17 (CWP) n = 19 (HC)	ACR 1990	Interstitial OEA, PEA, and SEA (trapezius muscle)	Microdialysis/liquid chromatography-tandem mass spectrometry (LC-MS/MS)	CWP: 48.8 (10.0) HC: 41.8 (10.7)	CWP: 0/17 HC: 0/19	FMS: 27.5 (5.7) HC: 24.5 (2.9)	Not reported	Not reported.	Not reported Subjects with bursitis, disorders of the spine, tendonitis, capsulitis, postoperative conditions (neck/shoulder), prior neck trauma, neurological disease, rheumatoid arthritis or any other systemic disease, metabolic disease, malignancy, severe psychiatric illness, and pregnancy were excluded	Pain (NRS 0–10): CWP vs HC: *P* < 0.001 Depression/anxiety: Not reported	Not reported	CWP (nM) 120 min: OEA: 2.22 (1.78) PEA: 0.93 (0.50) SEA: 3.67 (3.54)	HC (nM) 120 min: OEA: 0.34 (0.48) PEA: 0.55 (0.95) SEA: 0.74 (0.69)	*P*: OEA: 0.000 PEA: 0.148 SEA: 0.001	

2-AG, 2-arachidonoylglycycerol; ACR, American College of Rheumatology; AEA, N-arachidonoylethanolamide, or anandamide; AM, morning sample; CWP, chronic widespread pain; FMS, fibromyalgia syndrome; HCs, healthy controls; IL, interleukin; LEA, linoleoylethanolamide; OEA, oleoylethanolamide; PEA, palmitoylethanolamide; PM, afternoon sample; SEA, stearoylethanolamide; TNF-α, tumor necrosis factor-α.

### 3.1. Meta-analyses

Seven individual meta-analyses were performed to compare the differences in plasma and interstitial levels of endocannabinoids and NAEs between people with CWP and FMS and healthy controls. Table 3 contains a summary of statistics on the meta-analyses.

**Table 3:** Results of meta-analyses of plasma and interstitial levels of endocannabinoids and N-acylethanolamines in patients with fibromyalgia syndrome and chronic widespread pain.

Outcome	No. of reports	No. of subjects		SMD (95% CI)	*P* (overall effect)	Heterogeneity I^2^, χ^2^, *P*
		Case	Control			
Plasma AEA						
FMS vs HC	3	230	185	−0.05 (−0.25, 0.15)	0.61	0%, 1.77, 0.41
Plasma 2-AG						
FMS vs HC	3	230	185	0.22 (−0.09, 0.53)	0.16	52%, 4.20, 0.12
Plasma PEA						
FMS vs HC	3	230	185	0.15 (−0.14, 0.44)	0.31	46%, 3.73, 0.15
CWP vs HC	3	31	48	0.53 (0.01, 1.06)	**0.05**	17%, 2.40, 0.30
Total	6	261	233	0.24 (−0.02, 0.51)	0.07	37%, 7.98, 0.16
Plasma OEA						
FMS vs HC	2	141	149	0.33 (0.10, 0.57)	**0.005**	0%, 0.68, 0.41
CWP vs HC	3	31	48	0.44 (−0.47, 1.35)	0.34	70%, 6.77, 0.03
Total	5	172	197	0.37 (0.00, 0.73)	**0.05**	49%, 7.91, 0.09
Plasma SEA						
FMS vs HC	2	141	149	0.52 (0.28, 0.75)	**<0.0001**	0%, 0.02, 0.90
CWP vs HC	3	31	48	0.31 (−0.15, 0.77)	0.19	0%, 1.27, 0.53
Total	5	172	197	0.47 (0.27, 0.68)	**<0.00001**	0%, 1.90, 0.75
Interstitial PEA						
CWP vs HC	2	35	43	0.26 (−0.19, 0.71)	0.25	0%, 0.76, 0.38
Interstitial SEA						
CWP vs HC	2	35	43	0.86 (0.33, 1.38)	**0.001**	19%, 1.24, 0.27

Bold font indicates statistical significance (P ≤ 0.05).2-AG, 2-arachidonoylglycycerol; AEA, N-arachidonoylethanolamide or anandamide; CWP, chronic widespread pain; FMS, fibromyalgia syndrome; HCs, healthy controls; OEA, oleoylethanolamide; PEA, palmitoylethanolamide; SEA, stearoylethanolamide.

#### 3.1.1. Meta-analyses (x3) on plasma oleoylethanolamide, stearoylethanolamide, and palmitoylethanolamide in chronic widespread pain and fibromyalgia syndrome subgroups

**Figure 2.**
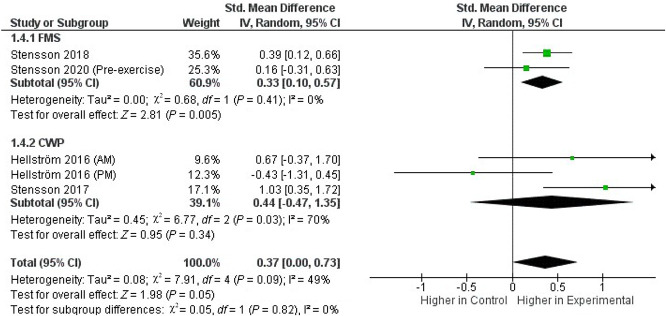
Forest plot of standardized mean difference (SMD) in plasma OEA levels in patients (FMS and CWP) and healthy controls. Weights obtained from random-effects analysis. Horizontal lines represent 95% CIs. The arrow indicates that the upper limit of the CI for that study is equal or superior to the upper limit of the SMD indicated at the bottom of the graph. The diamond shows the overall pooled SMD. The vertical line is the line of no effect, representing no difference between patients and healthy controls. CI, confidence interval; CWP, chronic widespread pain; FMS, fibromyalgia syndrome; OEA, oleoylethanolamide.

**Figure 3.**
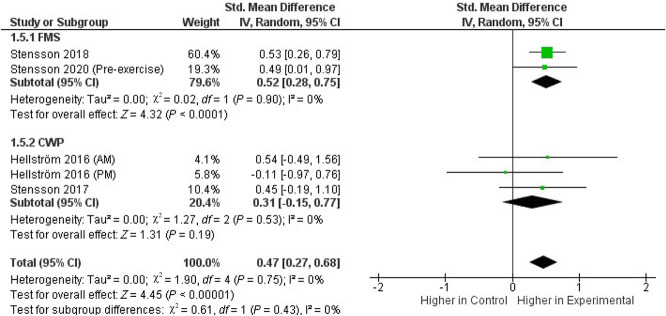
Forest plot of Standardized Mean Difference (SMD) in plasma SEA levels in patients (FMS and CWP) and healthy controls. Weights obtained from random-effects analysis. Horizontal lines represent 95% CIs. The arrow indicates that the upper limit of the CI for that study is equal or superior to the upper limit of the SMD indicated at the bottom of the graph. The diamond shows the overall pooled SMD. The vertical line is the line of no effect, representing no difference between patients and healthy controls. CI, confidence interval; CWP, chronic widespread pain; FMS, fibromyalgia syndrome; SEA, stearoylethanolamide.

We pooled the data from 2 FMS^54,57^ and 2 CWP studies^21,55^ that investigated OEA and SEA plasma concentrations. Across these 4 studies, which included in total 172 patients and 197 controls, the whole-group analysis demonstrated significantly higher concentrations of both OEA (Fig. 2) and SEA (Fig. 3) in the experimental group compared with those in healthy controls (SMD, 0.37; 95% CI, 0.00–0.73; *P* = 0.05 and SMD, 0.47; 95% CI, 0.27–0.68; *P* < 0.0001, respectively), with evidence of low heterogeneity (*I*^*2*^ = 49% and *I*^*2*^ = 0%, respectively). Increased plasma levels of both OEA and SEA were found in patients in the FMS subgroup (*P* = 0.005 and *P* < 0.0001, respectively), but not in the CWP subgroup (*P* = 0.34 and *P* = 0.19, respectively).

Three FMS and 2 CWP studies investigated plasma concentrations of PEA^4,21,54,55,57^ in a total of 261 patients and 233 controls. In the whole-group analysis, no significant difference was found in plasma PEA concentrations in the experimental group compared with those in healthy controls (*P* = 0.07), with evidence of low heterogeneity (*I*^*2*^ = 37%). Increased plasma PEA levels were found in patients in the CWP subgroup (*P* = 0.05), but not in the FMS subgroup (*P* = 0.31).

#### 3.1.2. Meta-analyses (x2) on plasma N-arachidonoylethanolamide and 2-arachidonoylglycerol in fibromyalgia syndrome

Three FMS studies reported data on plasma AEA levels and 2-AG levels, totaling 230 patients and 285 controls.^4,54,57^ No significant difference was found in plasma AEA concentrations in patients with FMS compared with those in healthy controls (SMD, −0.05; 95% CI, −0.25 to 0.15; *P* = 0.61; *I*^*2*^ = 0%). Similarly, no significant difference was found in plasma 2-AG concentrations in patients with FMS compared with those in healthy controls (*P* = 0.16), with evidence of moderate heterogeneity (*I*^*2*^ = 52%).

#### 3.1.3. Meta-analyses (x2) on interstitial palmitoylethanolamide and stearoylethanolamide in chronic widespread pain

Two CWP studies investigated interstitial levels of PEA and SEA in the trapezius muscle.^16,56^ One of these studies collected data before exercise (140 minutes after the insertion of the microdialysis catheter into the muscle) and after 20 minutes of standardized repetitive low-force exercise.^16^ Another study conducted the measurements at 20, 40, 60, 80, and 120 minutes after the insertion of the microdialysis catheter into the muscle.^56^ To ensure consistency between studies, we included the data before exercise from the first study^16^ and the data collected at 120 minutes from the second study.^56^ Across these 2 studies, which included 35 patients and 43 controls in total, significantly higher concentrations of SEA were reported in patients with CWP compared with those in healthy controls (SMD, 0.86; 95% CI, 0.33–1.38; *P* = 0.001), with evidence of low heterogeneity (*I*^*2*^ = 19%). No significant difference was found in interstitial PEA concentrations in patients with CWP compared with those in healthy controls (*P* = 0.25, *I*^*2*^ = 0%).

## 4. Discussion

Fibromyalgia syndrome is a syndrome of unknown etiology; although several theories have been proposed, the exact pathophysiology is still poorly understood.^30^ An extensive search for causality has led to the proposal of an endocannabinoid deficiency in the pathophysiology of FMS, suggesting that reduced ECS tone with decreased endocannabinoids and upregulated cannabinoid receptor activity might be involved in FMS.^47^ However, this hypothesis has not yet been supported by objective clinical data.

To our knowledge, this is the first systematic review and meta-analysis exploring the clinical relevance of ECS alterations in patients with CWP and FMS by comparing the differences in plasma and interstitial levels of endocannabinoids and NAEs between patients and healthy controls. Contrary to the endocannabinoid deficiency theory, the main findings identified increased plasma levels of OEA and SEA in people with FMS compared with those in controls and increased plasma levels of PEA and interstitial levels of SEA in people with CWP compared with those in controls. There were no significant differences in other ECS parameters, including plasma AEA and 2-AG levels, between patients with CWP or FMS and healthy controls.

The one major impediment to investigating the function of the endocannabinoids is their short lifetime.^31,64^ They are produced on demand and are rapidly metabolized by their degrading enzymes,^27^ making it difficult to study their effects in vivo and limiting their use as a therapeutic intervention. Recent advances have focused on developing compounds that inhibit the endocannabinoid degrading enzymes, thereby increasing their availability in pain-regulatory circuits.^27^

In addition, there are several peripheral factors that may affect endocannabinoid and NAE levels, including exogenous cannabinoids,^12^ inflammation,^2^ hypothalamic-pituitary-adrenal (HPO) axis activation,^26^ alterations of the gut microbiome and metabolic function,^8^ and medications.^60^ These peripheral factors may be associated with increased SEA, OEA, and PEA levels seen in patients with CWP and FMS in this review.

Nonopioid analgesics, such as NSAIDs, paracetamol, and dipyrone, may potentially elevate endocannabinoid levels through several mechanisms, including inhibition of endocannabinoid cellular uptake, stimulation of endocannabinoid biosynthesis and release, and inhibition of the activity of degradative enzymes (FAAH and COX-2).^60^ Because prolonged washout periods of analgesics and antidepressants in patients with chronic pain may be unethical and challenging to perform, most of the studies in this review instructed their participants to abstain from their medication only for 12 to 48 hours before the investigations, which could affect the results by affecting the levels of endocannabinoid and NAEs in the participants. Furthermore, the endogenous activity of the ECS is affected by exogenous cannabinoids, such as delta-9-tetrahydrocannabinol (THC) and cannabidiol (CBD), which directly or indirectly modulate levels of endocannabinoids by acting as either agonists or antagonists to the cannabinoid receptors and modifying endocannabinoid cellular reuptake and endocannabinoid enzyme activity.^12^ One publication included in this review excluded cannabis users from their study,^4^ whereas others did not provide any information on current or prior cannabis use among their participants. Future studies should use standardized measures to assess medication and cannabis use to clarify their association with levels of endocannabinoids and NAEs in people with CWP and FMS.

The ECS plays an important role in energy metabolism, with evidence suggesting a correlation between circulation endocannabinoids and body weight and adiposity. Higher plasma and saliva levels of endocannabinoids and NAEs have previously been reported in obese individuals than those of normal weight.^10,13,41^ In most studies included in this review, the BMI in participants in the experimental groups was higher than that in participants in the control groups, providing another potential confounder influencing the results. One study identified BMI as a covariate for plasma AEA and PEA levels in patients with FMS.^4^ Similarly, a study conducted by Stensson et al.^57^ found a significant positive correlation between plasma 2-AG levels and BMI in patients with FMS, while an earlier study by the same authors found no correlation between BMI and interstitial levels of OEA, PEA, and SEA in patients with CWP.

Endocannabinoid system activity is altered in many inflammatory, metabolic, psychological, and psychiatric disorders.^43,52,62,67^ Thus, significantly increased endocannabinoid levels have been found in several health conditions associated with systemic inflammation, including chronic hepatitis C,^45^ cirrhosis,^9^ pancreatitis,^46^ atherosclerosis,^39^ myocardial infarction,^63^ endometriosis,^50^ and repeated exposure to sunlight.^14^ Furthermore, acute or chronic exposure to physical or psychological stress may also increase endocannabinoid levels.^26^ Most of the studies in this review excluded individuals with conditions that would interfere with the outcomes, including diabetes, osteoarthritis, hypertension, malignancies, and other inflammatory, metabolic, psychiatric, and neurological conditions.^16,21,34,54–57^ One study identified diabetes mellitus as a covariate for plasma 2-AG levels in patients with FMS,^4^ while a study by Stensson et al.^57^ reported a significant positive correlation between plasma AEA levels and depression scores in patients with FMS. A study by Stensson et al.^56^ found no correlation between plasma levels of OEA, PEA, and SEA and inflammatory cytokines in patients with CWP.

In addition, endocannabinoids levels may change significantly throughout the day, depending on many factors, including satiety levels, distress, physical activity, and sleep quality.^25,58^ Recent studies have shown that circulating endocannabinoids exhibit significant circadian rhythmicity, where levels of 2-AG increased continuously across the morning, peaking in the early to mid-afternoon and were significantly enhanced by sleep restriction,^18^ whereas AEA, PEA, and OEA demonstrated a biphasic pattern with lesser amplitude peaking during early sleep and mid-afternoon and were not affected by sleep restriction.^17^ One study in this review attempted to correlate hours of sleep the previous night with plasma AEA, 2-AG, and PEA levels in patients with FMS; however, no such correlation was reported.^4^

Endocannabinoid levels are sensitive to several aspects of food intake. Research showed that circulating levels of AEA significantly increased before meal consumption and then decreased postprandially, whereas no meal-related changes were observed for 2-AG.^15^ The response of 2-AG to food seems to be affected by its perceived hedonic (pleasure-driven) value. Thus, circulating levels of 2AG increased before, during, and after consumption of favorite foods, but not after nonhedonic eating.^44^ In addition, prolonged consumption of high-fat diets may decrease endogenous levels of NAEs (ie, OEA and PEA) and encourage food overconsumption.^19^ Most studies in this review did not control or report dietary factors. One study reported time since last food intake as a covariate for plasma AEA and PEA levels in patients with FMS.^4^

Several studies have examined the effects of physical exercise on levels of endocannabinoids and NAEs, including 2 studies in this review, one of which explored plasma levels of AEA, OEA, PEA, SEA, and 2-AG before and after a 15-week resistance exercise program in patients with FMS,^54^ whereas the other studied interstitial levels of PEA and SEA in the trapezius muscle before and after 20 minutes of repetitive low-force exercise in patients with CWP.^16^ Owing to the limited number of studies, it was not possible to perform the quantitative synthesis of this data as part of this review. Nonetheless, the first study reported significantly increased AEA plasma levels in both female patients with FMS and healthy controls and significantly decreased SEA levels in patients with FMS after the 15-week exercise program.^54^ The second study demonstrated significantly lower interstitial levels of PEA and SEA in the CWP cohort postexercise.^16^

Many factors can affect ECS activity making it difficult to achieve reliable results without tight control measures during experiments to understand and mitigate effects from such factors on those results. Future studies should explore the correlation between endocannabinoid levels and comorbidities, stress levels, physical activity, sleep quality, circadian rhythm, and dietary factors in people with CWP and FMS.

Moreover, further research is required to investigate other factors that may influence ECS tone in people with CWP and FMS, including changes in the expression of CB_1_ and CB_2_ receptors and the activity of endocannabinoid synthesizing and degrading enzymes.

Investigating endocannabinoid activity in these conditions is particularly relevant because it provides the scientific basis for future translational research, ie, clinical trials that explore exogenous cannabinoids as novel therapeutics in FMS. Evidence of enhancing ECS tone by using exogenous cannabinoids (ie, CBD or THC) in individuals with FMS is limited but promising and, if initial results are confirmed, could represent a new therapeutic approach.^36^

### 4.1. Limitations

This systematic review and meta-analysis have several limitations. Firstly, all quantitative analyses of plasma and interstitial levels of endocannabinoids and NAEs were based on a small number of studies with relatively small samples sizes.

Secondly, most studies provided limited data on cannabis use, concomitant medication, comorbidities, physical activity, stress levels, circadian rhythm, sleep quality, and dietary factors, variables that may influence ECS function. Future studies should use standardized measures to assess these variables to clarify their association with levels of endocannabinoids and NAEs.

Thirdly, most of the studies investigated levels of endocannabinoids and NAEs in female-only cohorts. The number of diagnosed cases of FMS in men is relatively small; therefore, most research on the condition has been performed on women, often overlooking the study of FMS in men. Evidence suggests that sex differences exist in the function and expression of ECS components, potentially due to interactions between the ECS and hormones and differences in metabolism and cannabinoid receptor expression.^6^ Therefore, the role of sex in ECS function in CWP and FMS should be further explored in future studies.

Fourthly, most studies in this review were performed by a single research group in one geographic area, which likely limits the generalizability of the findings. Therefore, it is important to expand this research to a broader population.

## 5. Conclusions

This study investigated the prognostic and diagnostic potential of measuring plasma and interstitial levels of endocannabinoids and NAEs in patients with CWP and FMS. However, most of the studies did not account for important variables that may influence ECS function, including cannabis use, concomitant medication, comorbidities, physical activity, stress levels, circadian rhythm, sleep quality, and dietary factors. Owing to these limitations, the review was not conclusive as to whether plasma and interstitial levels of endocannabinoids and NAEs are clinically relevant in patients with CWP and FMS. Understanding endocannabinoid activity in CWP and FMS will provide the scientific basis for the use of exogenous cannabinoids as potential therapeutics in these disease states and underpin further translational research in the area.

## Disclosures

The authors declare the following financial interests/personal relationships, which may be considered as potential competing interests: L. N. Warne holds the position of Head of Research & Innovation at Little Green Pharma Ltd; I. Kurlyandchik holds a PhD scholarship, which is partially funded by Little Green Pharma Ltd. The other authors have no competing interests to declare.

## Appendix A. Supplemental digital content

Supplemental digital content associated with this article can be found online at http://links.lww.com/PR9/A177.
